# Chordoma: a case series and review of the literature

**DOI:** 10.1186/s13256-018-1784-y

**Published:** 2018-08-27

**Authors:** Ozkan Alan, Tugba Akin Telli, Ozlem Ercelep, Eda Tanrikulu Simsek, Tugba Basoglu Tuylu, Aydan Mutis, Rahib Hasanov, Serap Kaya, Nalan Akgül Babacan, Faysal Dane, Perran Fulden Yumuk

**Affiliations:** 1 Division of Medical Oncology, Department of Internal Medicine, Marmara University Faculty of Medicine, Istanbul, Turkey; 2 Department of Internal Medicine, Marmara University Faculty of Medicine, Istanbul, Turkey

**Keywords:** Advanced chordoma, Tyrosine kinases, Rare disease, Targeted therapy

## Abstract

**Background:**

Chordoma is a rare malignant tumor of the skull base and axial skeleton, with an incidence of less than 0.1/100,000 per year. Patients with advanced chordoma have a poor prognosis due to locoregional recurrence with infiltration and destruction of surrounding bone and soft tissue. Cytotoxic chemotherapy or other systemic therapies have not been proven to be effective for these diseases. Therefore, several molecularly targeted therapies have been proposed as potentially beneficial, including tyrosine kinase inhibitors such as imatinib, sorafenib, lapatinib, and others.

**Case presentation:**

We present three cases of advanced chordoma treated with molecular targeted therapies: a 52-year-old Caucasian man, a 72-year-old Caucasian woman, and a 38-year-old Caucasian woman.

**Conclusions:**

Chordoma has few systemic treatment options and they have limited benefit. Randomized trials with large patient numbers are unfeasible in this rare disease. Targeted therapy might be a reasonable alternative treatment for chordoma. Still, new treatment strategies are needed for this rare disease.

## Background

Chordoma is a rare malignant tumor of the skull base and axial skeleton, with an incidence of less than 0.1/100,000 per year [1]. The pathogenesis of chordoma remains undetermined. It is presumed that the notochord developed in fetal development evolved from cellular residues [2]. Patients with advanced chordoma have a poor prognosis due to locoregional recurrence with infiltration and destruction of surrounding bone and soft tissue. Cytotoxic chemotherapy or other systemic therapies have not been proven to be effective for these diseases. Therefore, several molecularly targeted therapies have been proposed as potentially beneficial, including tyrosine kinase inhibitors (TKIs) such as imatinib, sorafenib, lapatinib, and others. Due to the small number of cases in which TKIs are used as a treatment option, there are very limited data. We present three cases of advanced chordoma treated with molecular targeted therapies. This case series reports the stable response of patients with chordoma to the TKIs imatinib and sunitinib. In the Marmara University Medical Oncology Department, three patients with chordoma were treated with these agents between 2015 and 2017: one patient with imatinib, one patient with sunitinib, and one patient with imatinib and sunitinib (Table 1).

**Table 1:** Overview of patients

Patient	Localization	Prior local treatment	First-line systemic tx	Second-line systemic tx	Results
1	Sacrum	Surgery Radiotherapy	Imatinib 400 mg [10]	Best supportive care	PD 16 months
2	Clivus	Surgery Radiotherapy	Sunitinib 37.5 mg [14]		SD 20 months
3	Clivus	Surgery Radiotherapy	Imatinib 400 mg [10]	Sunitinib 37.5 mg [14]	Imatinib PD 25 months Sunitinib PD 6 months

*PD* progressive disease, *SD* stable disease, *tx* treatment

## Case presentation

### Case 1

A 52-year-old Caucasian man who had no medical history presented with sacral region pain that had continued for 3 months in April 2012. There was no significant family or psychosocial history. He did not take any medications. He was a computer engineer and his job scope was mainly office work. He lived with his wife and one child in a small flat in Istanbul. He was an active tobacco smoker with a 10 pack year smoking history. Currently, he smoked five cigarettes a day. He did not consume alcohol. His physical examination revealed tenderness and swelling in the sacral region. His vital signs were stable with blood pressure 125/65, pulse rate 70/minute, and temperature 36.2 °C. A systemic examination was normal and no neurological abnormality was detected. Vertebral magnetic resonance imaging (MRI) showed a pathological fracture in L5. After a Tru-Cut biopsy, he was diagnosed as having chondroid chordoma. He was treated with preoperative stereotactic radiotherapy to L5 vertebra at a total dose of 15 Gray in two fractions with CyberKnife followed by surgery in May 2012. We aimed to reduce surgical complications by preoperative stereotactic radiotherapy. Two years later, in May 2014, he presented with lumbosacral region pain and MRI suggested recurrent tumor involving L4, L5, and S1 vertebrae. He was not eligible for surgery and was treated with definitive radiotherapy with intensity-modulated radiotherapy (IMRT) mainly for palliative intent. Between 7 July 2014 and 8 August 2014, he received 40 Gray to L4, L5, and S1 vertebrae in 20 fractions 5 days a week for 4 weeks. In January 2016, he presented with paraplegia. Control imaging showed local recurrence, multiple lung nodules, and sternal metastasis. Imatinib 400 mg was started in February 2016 and continued until July 2017 when control imaging showed the progression of his disease. He received a total of 16 months of treatment. During this period, he received 400 mg/day of imatinib and no dose reduction was done. The laboratory results are given in Table 2**.** Overall, he tolerated treatment well and did not report any side effects. The best supportive care was offered. He was treated with best supportive care until May 2018. He died on 25 May 2018.

**Table 2:** Laboratory findings of cases

		Case 1	Case 2	Case 3
WBC (μ/*L*)	Pretreatment*	8900	9500	7650
	Posttreatment**	4300		4270
HGB (g/dl)	Pretreatment*	13.4	11.9	12.2
	Posttreatment**	12.1		10.9
PLT (μ/*L*)	Pretreatment*	245,000	342,000	267,000
	Posttreatment**	158,000		145,000
AST (U/L)	Pretreatment*	34	32	45
	Posttreatment*	53		67
ALT (U/L)	Pretreatment*	18	42	54
	Posttreatment**	39		83
Creatinine (mg/dl)	Pretreatment*	0.8	1.2	0.98
	Posttreatment*	0.91		1
Bilirubin total (mg/dl)	Pretreatment*	0.8	1	0.98
	Posttreatment**	0.89		1

*ALT* alanine aminotransferase, AST aspartate aminotransferase, *HGB* hemoglobin, *PLT* platelet, *WBC* white blood cell, * before the start of the tyrosine kinase inhibitor, ** after the discontinue of tyrosine kinase inhibitor therapy. Case 2 treatment continues

### Case 2

A 72-year-old Caucasian woman who had type 2 diabetes and hypertension presented with diplopia in February 2010. Her vital signs were abnormal. Her blood pressure was high (150/95 mmHg), and her pulse rate and temperature were 65/minute and 37.1 °C. A neurological examination showed preserved muscular and neurological function and no signs of paresthesia or hypoesthesia; a general examination showed no other abnormality. There was no significant family or psychosocial history. She was taking perindopril 10 mg/day, metformin 2000 mg/day, and nateglinide 360 mg/day. She was a housewife and lived with her husband in a small town. She never smoked tobacco and did not consume alcohol. A brain and sella MRI showed a 3 cm x 2 cm x 2 cm mass in the sellar and parasellar region. She was operated on via transsphenoidal surgery. A postoperative pathology examination revealed chordoma. After the surgery, gamma-knife radiotherapy was performed. She came back in March 2014 and a 12 mm × 30 mm clivus mass was revealed on her brain MRI. She was operated on again and a pathology examination revealed chordoma. Postoperative stereotactic radiotherapy to residual mass in her clivus at a total dose of 12 Gray in one fraction with gamma-knife was done. Two years later, she had a recurrent mass in her clivus. As neither further surgery nor radiotherapy were suitable for her, sunitinib 37.5 mg per day was started in April 2016 and she has been receiving the same treatment ever since. The laboratory results are given in Table 2. She reported intermittent grade 1 nausea and grade 1 fatigue; no serious side effects were reported. The best response to sunitinib treatment was assessed as stable disease. In June 2018, she continues with the same dose of treatment. There is no detected progression of her disease.

### Case 3

**Fig. 1:**
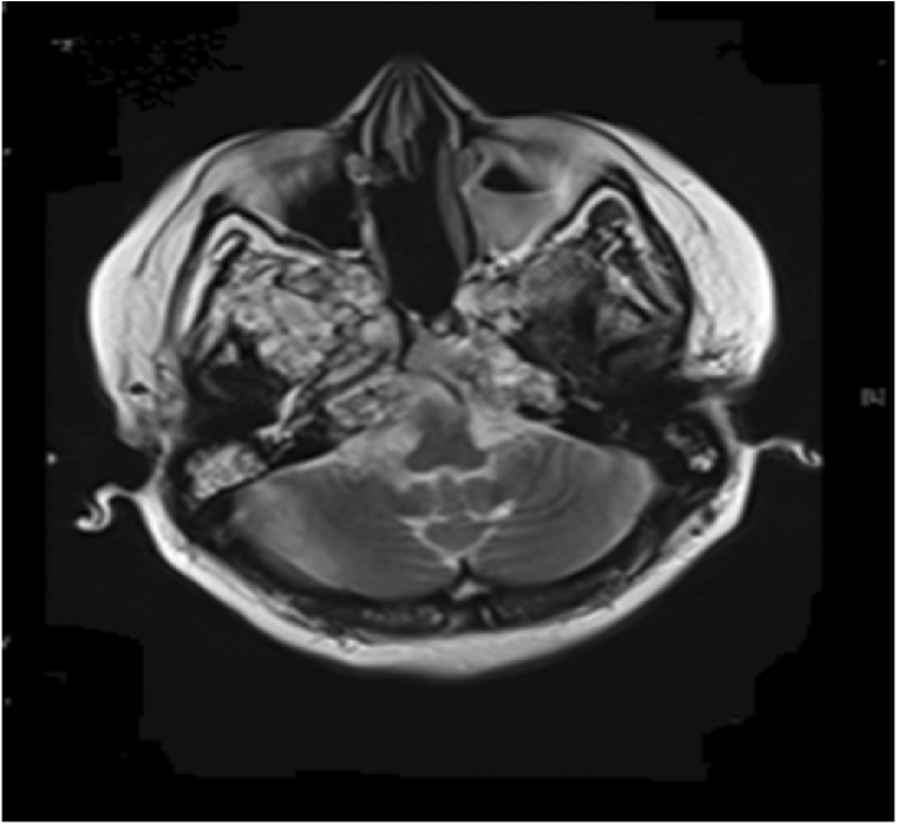
Magnetic resonance image of patient 3 treated with imatinib (progressive disease 25 months)

**Fig. 2:**
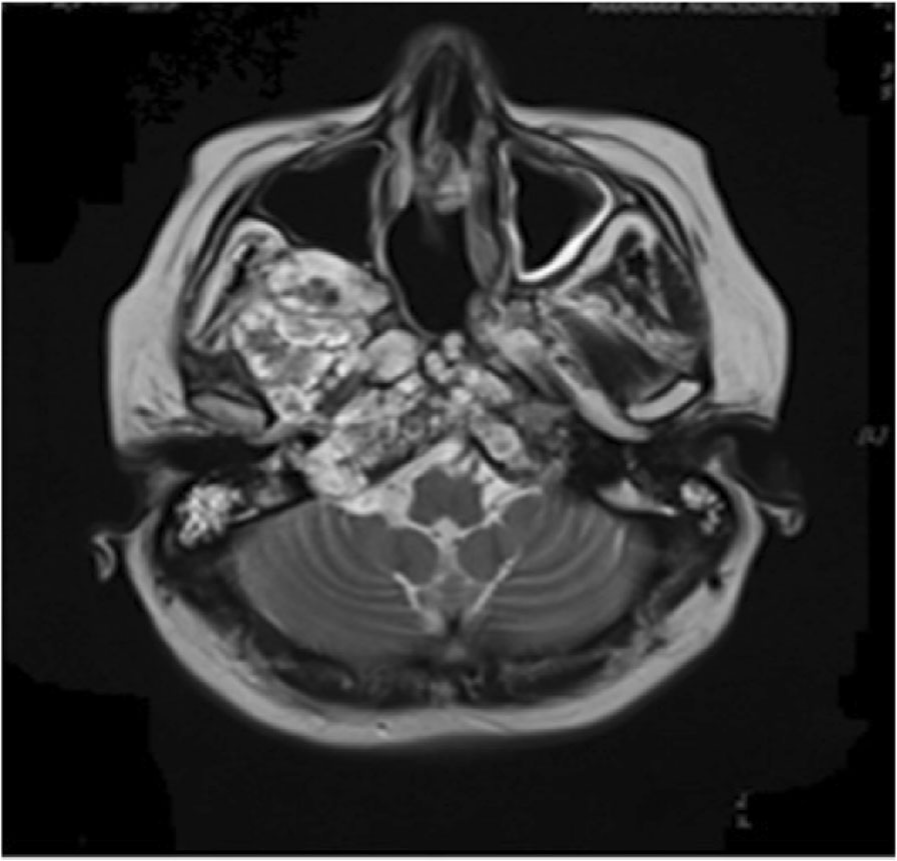
Magnetic resonance image of patient 3 treated with sunitinib (progressive disease 6 months)

A 38-year-old Caucasian woman who had no medical history presented with a headache of 2 months’ duration in August 2012. There was a family history of malignancies. She had no psychosocial history. She did not take any medications. She was a housewife. She lived with her husband and three children in a flat in the city center of Istanbul. She never smoked tobacco and did not consume alcohol. Her vital signs were stable with blood pressure 110/70 mmHg, pulse rate 82/minute, and temperature 36.5 °C. On neurological examination, there was a limitation of temporal movement in her right eye. There were no signs of paresthesia or hypoesthesia. A general examination showed no other abnormality. Brain MRI showed 34 mm × 10 mm and 20 mm × 19 mm masses in her clivus. She was operated on and a pathology examination showed chordoma. Postoperative stereotactic radiotherapy to clivus at a total dose of 24 Gray in one fraction with gamma-knife was performed. In March 2014, she had a recurrence in her clivus and then she underwent another operation. A pathology examination revealed chordoma. She presented with diplopia for 1 month in January 2015. Brain MRI detected a recurrent mass in her clivus and invasion to the pons. She was treated with external cranial radiotherapy for palliative intent. She received a total of 30 Gray to recurrent mass in ten fractions during 10 days. In March 2015, a residual mass in her clivus was seen in MRI. She was started on daily 400 mg of imatinib in April 2015. The best response to imatinib was stable disease**.** Overall, imatinib was well tolerated; she reported periorbital edema, grade 1 skin rash on her legs, and nausea grade 2. She complained of visual loss in her left eye in May 2017. Brain MRI confirmed progressive disease (Fig. 1). Imatinib was stopped and sunitinib 37.5 mg per day was started in June 2017. She received sunitinib until December 2017 when she had radiological and clinical progression (Fig. 2). Four weeks later, her sunitinib dose was lowered to 25 mg/day due to ongoing grade 2 nausea and vomiting. Other reported symptoms included grade 2 fatigue and grade 1 hand-foot syndrome. The laboratory results are given in Table 2. She received a total of 25 months of imatinib therapy and 6 months of sunitinib treatment. She continued her follow-up with best supportive care until April 2018. She died on 23 April 2018.

## Discussion

We presented three patients whose tumor progressed after local treatment and who then received systemic tyrosine kinase treatment. Systemic tyrosine kinase treatment plays an important role in the treatment of recurrent chordoma. The tumors of cases 1 and 3 progressed during the treatment. Case 2 continues to be treated with sunitinib. The best radiological response was evaluated as stable disease. No radiological regression response was detected according to Response Evaluation Criteria in Solid Tumors (RECIST) criteria. All of the patients reported no significant side effects during treatment. Treatment was well tolerated.

Chordoma is a primary bone tumor originating from a non-differentiated notochordal residue and developed in the vertebrae; it is most frequently seen in sacral (50%), skull base (30%) and mobile spine (20%) [3]. The pathogenesis of chordoma is unclear but tumor cells are characterized by a notochordal differentiation. Chordoma is more frequent in men than in women. The average age at diagnosis is approximately 60 years, while the skull base presentation affects teenagers and children. Three subtypes have been described as pathological. The classical form and chondroid form are generally low grade and locally aggressive tumors, but the dedifferentiated form shows aggressive behavior [2]. Stacchiotti and Sommer published the first guidelines for the diagnosis and treatment of chordoma in 2015 [3]. Complete surgical resection with negative surgical margin in localized disease is the mainstay of care. Standard adjuvant radiotherapy is recommended for cervical spine and skull base chordoma. Definitive radiotherapy is the option in cases where resection is not suitable. Radiotherapy is recommended after R1 resected sacral chordomas. On the other hand, another retrospective study showed that local progression-free time is longer with the addition of radiotherapy [4]. Ten-year local progression-free survival (PFS) was 35-50% in patients with sacral chordoma who treated by adjuvant radiotherapy [3]. Twenty-nine patients with chordomas of the mobile spine and sacrum who were treated by surgery and high-dose proton−/photon irradiation were evaluated in a phase II trial [5]. In this trial, no significant difference between R0 and R1/R2/biopsy could be shown regarding local control [5]. Retrospective data of 17 patients compared surgery only with carbon ion therapy. The local recurrence-free survival rate at 5 years was 62.5% for the surgery group and 100% for the carbon ion radiotherapy group, and the disease-specific survival rate at 5 years was 85.7% and 53.3%, respectively [6]. Metastases can occur in 30–40% of the patients and that usually occurs after local relapse and at a later stage of the disease [7].

Cytotoxic chemotherapy does not have any proven benefit and therefore it is not recommended [3]. Tamborini *et al.* showed that beta-type platelet-derived growth factor receptor (PDGFRB) was highly expressed and phosphorylated, but platelet-derived growth factor receptor alpha (PDGFRA) and KIT were less expressed in chordomas [8]. Likewise, Weinberger *et al.* found epidermal growth factor receptor (EGFR) expression in a series of 12 patients with chordoma [9]. Therefore, in the last decade, molecular targeted therapy has been investigated for systemic therapy (Table 3). Imatinib, a platelet-derived growth factor receptor (PDGFR) TKI has shown positive results in a phase 2 study in advanced chordoma. This trial included 56 patients. One patient (2%) achieved a partial response and 11 patients had a minor response. Thirty-five patients had stable disease (62.5%) and clinical benefits were reported for 64% of patients. Median PFS and overall survivals were 9 months and 35 months, respectively [10]. Another retrospective trial confirmed imatinib activity with 34 (74%) patients having stable disease [11]. Lapatinib in EGFR-positive chordomas has been investigated in a prospective phase 2 trial. The overall response rate was 33.3% with a median PFS of 8 months [12]. Sorafenib was given to 27 patients with chordoma in a phase 2 study [13]. Results showed 9 months PFS rate of 73% and 1-year overall survival rate of 86.5%. The activity of sunitinib was assessed in a basket trial in advanced sarcoma. Nine patients with advanced chordoma were treated. No objective responses were seen and four stable diseases were detected. Median PFS was 12 months [14]. There was a retrospective study by the French Sarcoma Group of 80 patients with advanced chordoma who were treated with first-line molecular targeted therapies [15]. Patients were treated with imatinib (77.5%), sorafenib (13.7%), erlotinib (6.3%), sunitinib (1.2%), and temsirolimus (1.2%). Five patients had partial response (three patients treated imatinib, one with sorafenib, and one with erlotinib), and 58 patients had stable disease (72.5%). Median progression-free and overall survivals were 9.4 months and 52.8 months [15]. A retrospective case series of five patients with advanced chordoma was published. Four patients were treated with pazopanib and one patient was treated with sunitinib. Median PFS was 8.5 months in the pazopanib subgroup, and 27 months in the sunitinib subgroup [16].

**Table 3:** Survival data with prospective phase 2 trials and retrospective series

Phase 2 Trials				Retrospective series		
Treatment	Lapatinib [12]	Sorafenib [13]	Imatinib [10]	French Sarcoma Group [15]	Imatinib [11]	Pazopanib and sunitinib [16]
Number of cases	18	27	56	80	48	5
PFS (months)	8.2	NR	9	9.4	9.9	14
OS (months)	25	NR^*^	34.5	52.8	30	-
BORR (%)	0	3.7	2	6	0	20 (one patient)

*BORR* best objective response rate, *NR* not reached but > 15 months, *OS* median overall survival, *PFS* median progression-free survival, * 12 months OS rate 86.5%

We presented three cases of advanced chordoma. Cases 1 and 3 were treated with first-line imatinib: PFS 16 months and 25 months respectively. Case 2 was treated with first-line sunitinib and stable disease was evaluated. She continues to receive treatment. After tumor progression with imatinib, Case 3 treated with sunitinib (PFS was 6 months). None of our cases achieved a partial response. The best response of the three cases to TKIs treatment was assessed as stable disease. Before the systemic therapy, the patients were treated with local treatment (surgery and radiotherapy). The results of local treatments were evaluated: median PFS were over 2 years. Post-local treatment PFS was longer than post-TKI treatment PFS.

## Conclusions

Treatment planning in chordoma is challenging when local therapy is not an option after several relapses. Chordoma has few systemic treatment options with limited benefit. Randomized trials with large patient numbers are unfeasible in this rare disease. Targeted therapy might be a reasonable alternative treatment for chordoma. However, as in our case series, the best response with targeted therapy in the literature is stable disease. Although treatment options for patients with recurrent chordoma are increasing nowadays, chordoma causes severe morbidity and mortality. Therefore, still, new treatment strategies are needed for this rare disease.
